# Epidemiology of Skin Cancer: Role of Some Environmental Factors 

**DOI:** 10.3390/cancers2041980

**Published:** 2010-11-24

**Authors:** Gabriella Fabbrocini, Maria Triassi, Maria Chiara Mauriello, Guglielma Torre, Maria Carmela Annunziata, Valerio De Vita, Francesco Pastore, Vincenza D’Arco, Giuseppe Monfrecola

**Affiliations:** 1 Department of Systematic Pathology, Division of Dermatology, University of Naples Federico II, Naples, Italy; E-Mails: mcm86@live.it (M.C.M); crazymarica@hotmail.com (M.C.A.); valeriodevita@yahoo.it (V.D.V.); frankpast@libero.it (F.P.); enza.darco@hotmail.it (V.D.A.); monfreco@unina.it (G.M.); 2 Department of Preventive Medical Sciences, Division of Hygiene, University of Naples Federico II Naples, Italy; E-Mails: triassi@unina.it (M.T.); guytorre@fastwebnet.it (G.T.)

**Keywords:** skin cancer epidemiology, environmental factors, UV radiation, arsenic, climate change

## Abstract

The incidence rate of melanoma and non-melanoma skin cancer entities is dramatically increasing worldwide. Exposure to UVB radiation is known to induce basal and squamous cell skin cancer in a dose-dependent way and the depletion of stratospheric ozone has implications for increases in biologically damaging solar UVB radiation reaching the earth’s surface. In humans, arsenic is known to cause cancer of the skin, as well as cancer of the lung, bladder, liver, and kidney. Exposure to high levels of arsenic in drinking water has been recognized in some regions of the world. SCC and BCC (squamous and basal cell carcinoma) have been reported to be associated with ingestion of arsenic alone or in combination with other risk factors. The impact of changes in ambient temperature will influence people’s behavior and the time they spend outdoors. Higher temperatures accompanying climate change may lead, among many other effects, to increasing incidence of skin cancer.

## 1. Introduction

Melanoma, basal and squamous cell carcinoma represent the most common type of skin cancer in fair-skinned populations worldwide. The incidence rate of these types of tumor are dramatically increasing, whereas the mortality rate shows a stable or decreasing trend worldwide.

The purpose of this article is to provide a comprehensive review of epidemiology, incidence, etiology and related risk factors of skin cancer. A better understanding of the etiological factors is an essential step in the prevention of skin cancer. Ultraviolet radiation (UVR) derived from sun exposure is well-known to be the most important cause of skin cancer. Sunburn and excessive exposure to sun and tanning lamps are responsible for cumulative damage, which induces immunosuppression that is involved in the pathogenesis of skin cancer. Ozone depletion, levels of UV light, latitude, altitude, and also weather conditions, influence the emission of UV radiation reaching the earth’s surface. Moreover, environmental pollutants, chemical carcinogens and occupational exposures to carcinogens have been related to skin cancer. Finally, exposure to Chinese proprietary medicines, consumption of drinking water containing inorganic arsenic, skin color, and smoking are additional risk factors.

## 2. Ozone Depletion

**Figure 1:**
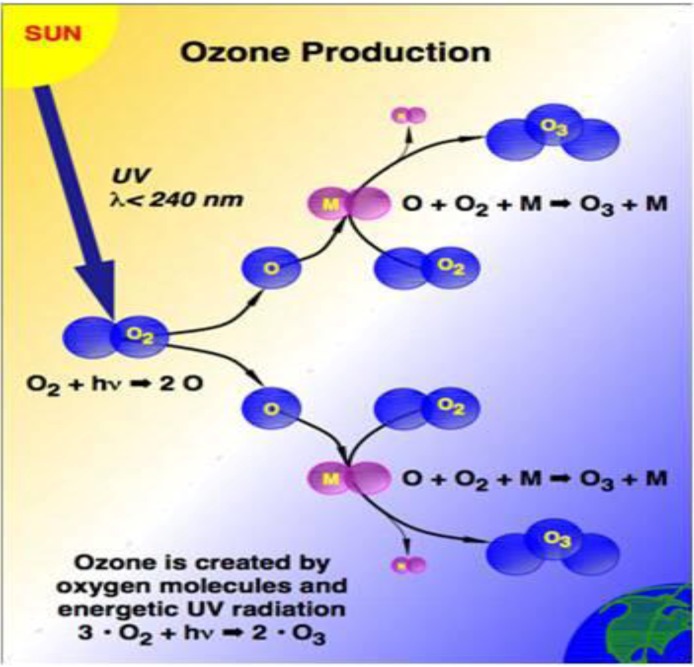
Ozone Production. Ozone is created by oxygen molecules and energetic UV radiation [9].

**Figure 2:**
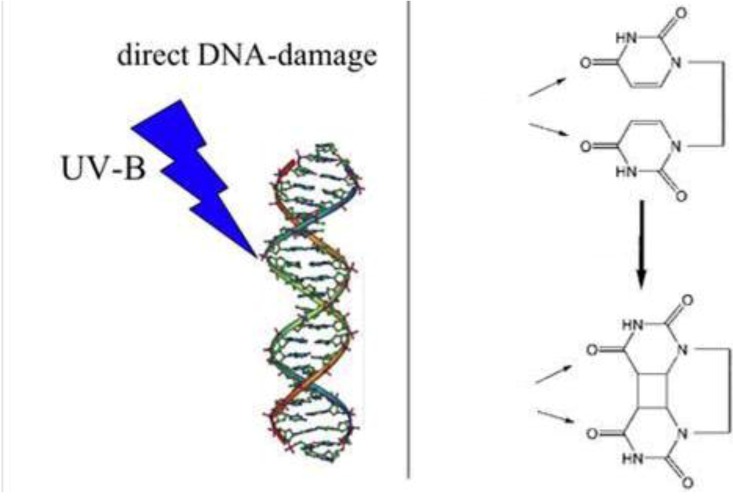
UVB DNA damage. UVB radiation may lead to direct DNA damage, inducing the development of thymine dimers, whereby adjacent thymine bases bond with each other instead of across the DNA backbone ladder. This thymine dimer makes a bulge, and the distorted DNA molecule does not function properly [10].

Ultraviolet-B-radiation (UVB, 280–320 nm) is well-known as an agent inducing non-melanoma (basal and squamous cell) skin cancer in a dose-dependent way. The effects of UVB exposure are enhanced by ozone depletion, which is responsible for an increase in biologically damaging solar UVB reaching the earth’s surface (Figure 1 and Figure 2) [1].

Stratospheric ozone depletion is most evident in polar regions, because of the vagaries of climate and weather. Nowadays, although some UVB impacts on human health are recognized, much is unclear and uncertain, and the effects of solar UVB radiation in these regions cannot be predict with certainty [2].

The increased incidence of skin cancer gives cause for concern all over the world. In white populations, epidemiological studies have revealed a correlation between an increase of skin cancer incidence and exposure to UV radiation. In fact, this increase was predominantly recorded in Caucasians living near the equator. Recent data assess the incidence of non-melanoma skin cancer in the U.S. at about 232/100,000, whereas, in Queensland (Australia) numbers as high as 2398/100,000 males and 1908/100,000 females have been reported [3]. Nowadays, despite many prevention and early diagnosis education programs, Australia has the world’s highest incidence rate of skin cancer. Therefore, melanoma and non-melanoma skin cancer represent a serious medical problem, both in terms of prevention and health costs [4]. This increased incidence seems to be the result of an “unnatural displacement” of fair-skinned populations to sub-tropical regions [5] and it is strongly correlated with geographical, environmental and social factors, such as high levels of UV enhanced in recent times by ozone depletion and the increase in time spent outdoors, encouraged by relatively cool summer temperatures [6].

An example of a sun-sensitive population, repeatedly exposed to high levels of UVB radiation, is represented by people who live in Punta Arenas (Chile), the southernmost city in the world (53 degrees S), located near the Antarctic ozone hole (AOH). This population (154,000) has been regularly exposed to an altered solar UV spectrum each spring for the last 20 years. In order to obtain *in situ* measurements of ozone and UVB radiation, a surveillance study had been performed in Punta Arena from 1987 to 2000. Data were collected by using a Brewer Spectrophotometer, that recorded a 56% reduction in stratospheric ozone (ozone levels as low as 145 DU: Dobson Units) and UVB levels up to 4.947 J/m^2^.

Residents were investigated for the presence of skin cancer: 19% of the total cases were recorded as cutaneous malignant melanoma (CMM) (incidence increased by 56%), whereas non-melanoma skin cancer (NMSC) represented 81% of the total (incidence increased by 46%).

Moreover, patients affected with CMM and NMSC had skin phototypes I-II in 59% and 54% of cases, respectively [7].

Changes in skin cancer incidence may be considered as a two-stage process, in which the increase in biologically effective UVB and the percentage increase in skin cancer incidence are involved. The first one results from an ozone loss of 1% (optical amplification factor, OAF), whereas the second one results from a 1% increase in annual UV dose (biological amplification factor, BAF). According to epidemiological data, the increased carcinogenic impact of UV radiation around 300 nm results in a value for OAF of approximately 1.6%, while the estimated values of BAF are approximately 1.7 for basal cell carcinoma and 3.0 for squamous cell carcinoma. These data suggest that a 10% decrease of ozone would eventually be sufficient to increase the incidence of basal and squamous cell carcinomas by almost 30% and 50%, respectively [8].

Furthermore, the increased skin cancer incidence has been related to changes in leisure time habits with increasing time spent outdoors and, accordingly, in UVB exposure [3].

## 3. Relationship between Surface UV Radiation and Air Pollution

Study of the relationship between surface UV radiation and the content of air pollutants was performed in Beijing using the radiative transfer model TUV4.4 (Tropospheric Ultraviolet Visible). Data collected from this study show that the average total ozone content is higher in seasons like winter and spring, and it is lower in summer and autumn. On the contrary, an inverse relationship exists between the average total ozone content and ground levels of UV radiation.

Further data show a reduction of more than 50% in the UV radiation on days with high levels of air pollution. In conclusion, the results of the study suggest that in Beijing, a correlation exists among the significant reduction in the UV radiation reaching the ground, the increased tropospheric ozone levels and nitrogen oxides [11].

A study performed by the North Caroline State University (USA) shows that specific air pollutants, such as black carbon and PM10 concentrations, can reduce the increase in the surface levels of UV radiation and thus offers an explanation for why—in spite of the stratospheric ozone depletion—surface UV increases have not been observed, especially in urban regions. The reasons for this apparent contradiction are the increased anthropogenic emissions that mask the decreases in stratospheric ozone. Precisely, black carbon can reduce the Diffey-weighted UV ground levels by as much as 35%, depending on the season [12], whereas at a low pollution Southern Hemispheric sub-tropical site (27.80 degrees S), the decrease in cloud cover is in part responsible for the ozone deficiency and consequently for the increased UVB radiation [13].

Moreover, although UVA radiation is indeed less erythrogenic and carcinogenic than UVB, it is proven to enhances skin cancer induced by UVB radiation. In fact, UVA combined with environmental pollutants (including also cigarette smoke) significantly increases the risk of skin cancer. Environmental pollutants, such as benzo[a]pyrene (BaP), are considered as photosensitizers that, when exposed to UVA radiation, can generate reactive oxygen species (ROS) [14].

## 4. Effects of the Pollutant Arsenic

Arsenic is a chemical element. It is a semi-metal that comes in three different allotropic forms: yellow, black and gray. The pure arsenic is not poisonous, but all its compounds that are used as pesticides, herbicides and insecticides are. In fact, arsenic is a very harmful environmental contaminant [15], and in humans, this element, is known to cause skin cancer [16] as well as cancer of the lung, bladder, liver, and kidney [17,18,19]. Millions of people are at risk of cancer and other diseases due to chronic exposure to this element [20,21].

Inorganic arsenic, a metalloid, is ubiquitously distributed in nature. In natural deposits, this metalloid forms a complex with pyrite, for which it has a strong affinity [22]. However, under certain conditions (pH, temperature, *etc*.), inorganic arsenic easily dissociates from its soil-bound forms and enters the aquifer [23]. For this reason, the major source of human exposure to arsenic is the drinking water of course, contaminated groundwater from wells. Exposure to high levels of arsenic in drinking water has been recognized for many decades in some regions of the world including China, India (Bangladesh and Bengal in particular), Taiwan and several countries in Central and South America [24,25].

**Figure 3:**
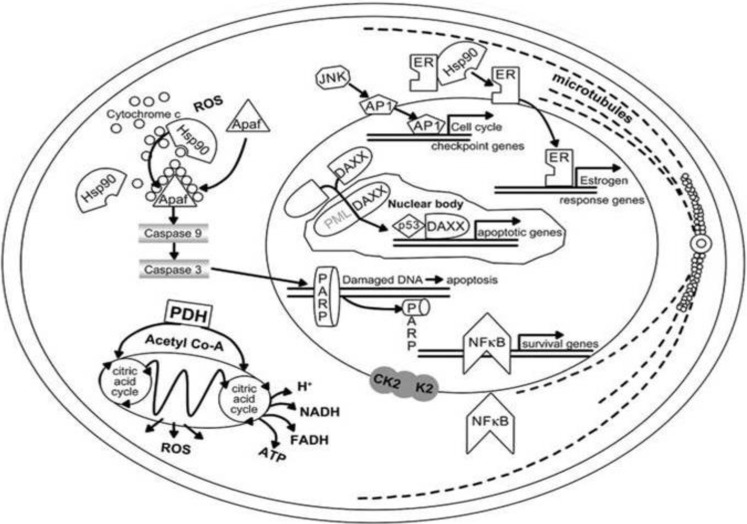
Cellular targets of arsenic trioxide action, with multiple pathways in malignant cells resulting in apoptosis or in the promotion of differentiation. Potential molecular targets for arsenic trioxide and arsenite are shown in gray. Abbreviations: AP1, activator protein-1; Apaf, apoptotic protease-activating factor; CK2, casein kinase; Co-A, coenzyme A; DAXX, death-associated protein; ER, estrogen receptor; FADH, flavin adenine dinucleotide; PARP, poly-(ADP-ribose)-polymerase; PML, promyelocytic leukemia. Modified from Miller *et al*. (32) with permission from the American Association for Cancer Research.

The carcinogenic potential of inorganic arsenic exposure through drinking water is a cause for considerable concern, especially because the hazardous inorganic arsenic is a powerful human multi-site carcinogen [25,26]. For example, in combination with UVB arsenic can cause skin cancer. Indeed Arsenic-UVB interaction provides a reasonable explanation for the rare cases of arsenical cancer in the sun-exposed skin. Multiple and recurrent skin lesions are associated with cellular immunity dysfunction in chronic arsenism [27]. In fact, arsenic treatment enhances the cytotoxicity, mutagenicity and clastogenicity of UV in mammalian cells, inducing apoptosis in keratinocytes by signals from caspase-9 and caspase-8, respectively (Figure 3).

The combination of UVB-Arsenic treatments results in antiproliferative and proapoptotic effects by stimulating both caspase pathways in keratinocytes. Inhibition of the expression of mutant p53 and Ki-67 produced by UVB irradiation results in an increased number of arsenic-induced apoptotic cells in Bowen’s disease lesions which caused an inhibitory effect on proliferation.

As for the arsenic compounds, apoptosis induction caused by As_2_O_3_ has been shown to be correlated with changes in intracellular calcium concentration [28,29]. The intracellular Ca^2+^ level increases immediately after adding As_2_O_3_. It has been suggested, in this regard, the opening of the mitochondria-dependent apoptotic pathway [30,31].

A precise cellular Ca^2+^-level regulation is also required for optimal DNA repair processes, DNA replication and gene expression. Arsenicals are able to modulate these processes. A direct correlation between arsenical’s genotoxic effects and disturbances of intracellular calcium concentration has been shown, but only partially and requires further investigations [24].

The association between arsenic ingestion and skin cancer has been documented since the late nineteenth century. Some of the earlier reports came from studies on patients treated with arsenical medications. Among the numerous studies conducted in the past century, much information about the types of abnormal cells came from case reports. SCC (Squamous Cell Carcinoma) has repeatedly been reported to be associated with the ingestion of arsenic alone or in combination with other risk factors. Also, reports on BCC (Basal Cell Carcinoma) related to arsenic ingestion are quite common. In addition to SCC and BCC, Bowen’s disease is often reported to be associated with arsenic ingestion, which might come from both drinking water and medication [32]. Several authors [32,33,34] also reported many cases of several skin diseases related to arsenic-containing medicine injections.

Molecular mechanisms could explain how arsenic acts, but the direct effect is yet unknown [18,35]. The main proposed mechanisms regarding arsenic carcinogenicity are induction of chromosomal abnormalities, promotion, and oxidative stress [18,36].

## 5. Climate Change and Human Skin Cancer

Climate change is a reality. Today, our planet records the highest temperatures and by the end of the century—if the current trends continue—the global temperature will probably reach the highest peak of the last two million years.

The changes in average temperatures will be accompanied by an increased frequency of extreme temperature events and an increased frequency of high summer temperatures.

Rising temperatures, accompanying climate change [37], may lead—among many other effects—to increasing incidence of skin cancer in human populations. It is not easy to determine the influence of temperature from data on skin cancer incidence in human populations residing in different regions. UV radiation is clearly the predominant factor and temperature is the second one. However, UV radiation and temperature are globally climatologically related. Going towards the equator, both the UV irradiance and temperature tend to increase. This general correlation makes it difficult to separate UV from the possible contribution of temperature [38]. The possibility that rising temperatures due to global warming could amplify the induction of skin cancer by solar UVR has been considered [39,40,41] and by van der Leun *et al*. [38], who suggested that long-term elevation of temperature by 2 °C as a consequence of climate change might increase the carcinogenic effectiveness of solar UV by 10%.

The impact of temperature change will influence people’s behavior and the time they spend outdoors. If such higher environmental temperatures in summer due to global warming are combined with drier weather, people living in middle latitudes may spend more time outdoors, thus increasing their solar UV exposure. Indeed, it has been shown, at least in school children in the U.K., that climate and temperatures influence behavior and, hence, sun exposure more than solar UV [42]. While this behavioral adaptation may have benefits in terms of vitamin D synthesis, the impact on skin cancer incidence and other health aspects of solar UVR exposure are expected to be negative. In a behavioral study in Australia [42,43], it was observed that the likelihood of sunburn approximately doubles when the temperature is 19–27 °C compared to temperatures of 18 °C or lower (currently typical average maximum summer temperature in the U.K.). The reason for this is probably because warmer temperatures encourage people to spend more time in direct sunlight, with the increased risk of sunburn. Interestingly, the study found that at temperatures above 27 °C, the risk of sunburns fall again as people sought shade for comfort reasons. In addition, there would be regional differences in behavioral responses to warming.

In conclusion, whether in nature the effect is physiological or not, the evidence for an effect of ambient temperature on human skin cancer incidence suggest a possibly substantial effect may come with temperature changes and climate change [38].

## 6. Conclusion

Climate changes, exposure to UVB and high levels of arsenic in drinking water, as well as several other environmental factors, have been reported to be associated with melanoma, SCC and BCC. As the incidence rate of melanoma and non-melanoma skin cancer is dramatically increasing worldwide, a clearer understanding of causative factors is an essential step in their prevention. Unfortunately, despite a large body of knowledge on skin carcinogenesis, previous studies have failed to individuate all the environmental risk factors for skin cancer. Therefore, further studies are required in order to investigate the potential effect of other possible risk factors and actuate prevention strategies based on avoiding them.
